# Blood cadmium is elevated in iron deficient U.S. children: a cross-sectional study

**DOI:** 10.1186/1476-069X-12-117

**Published:** 2013-12-30

**Authors:** Monica K Silver, Betsy Lozoff, John D Meeker

**Affiliations:** 1 Department of Environmental Health Sciences, University of Michigan School of Public Health, Ann Arbor, Michigan, USA; 2 Center for Human Growth and Development, University of Michigan, Ann Arbor, Michigan, USA

**Keywords:** Cadmium, Iron deficiency, Anemia, Serum ferritin, Free erythrocyte protoporphyrin, Transferrin saturation, NHANES, CDC

## Abstract

**Background:**

Cadmium (Cd), a widespread environmental contaminant, and iron deficiency (ID), the most common nutrient deficiency in the world, are known risk factors for neurodevelopmental delays, as well as other disorders, in infants and children. Studies assessing the cumulative effects of these factors are lacking in children, despite concerns of increased uptake of metals in the presence of ID. Here we sought to determine if blood and urine Cd levels were elevated in ID children compared to non-ID children.

**Methods:**

Data for 5224 children, aged 3–19 years, were obtained from the 1999–2002 NHANES. ID was defined as ≥2 of 3 abnormal iron indicators (low serum ferritin [SF], high free erythrocyte protoporphyrin [FEP], low % transferrin saturation [TSAT]); ID anemia (IDA) was defined as ID plus low hemoglobin (Hgb). Logistic regression was used to evaluate associations between ID, IDA, and abnormal iron indicators and categories of blood and urine Cd.

**Results:**

Adjusted odds of ID, IDA, low SF, and low TSAT were associated with increasing category of blood Cd but not urine Cd. Adjusted ORs (95% CI) for blood Cd ≥0.5 μg/L versus < LOD were = 1.74 (1.30-2.34), 4.02 (1.92-8.41), 4.08 (2.36-5.89) and 1.78 (1.32-2.39), for ID, IDA, low SF, and low TSAT, respectively. Age and sex specific analyses of blood Cd and ID/abnormal iron indicators revealed that the observed associations were strongest in females aged 16–19 years.

**Conclusions:**

Given their shared neurotoxic effects in children, and that many people live in areas with high burdens of both ID and Cd, more research into the complex relationships between nutrient deficiencies and environmental toxicants is vital.

## Background

Cadmium (Cd) is a toxic heavy metal that naturally exists in the earth’s crust. It has non-corrosive properties that make it desirable for use in a number of industrial products and processes, such as batteries, metal coatings, pigments, and plastics [1]. Non-occupationally exposed adults and children are exposed to Cd largely through the diet, with secondary exposures occurring from cigarette smoking or secondhand tobacco smoke, house dust, and industrial emissions [1,2]. Recent reports have also found that children may be exposed to Cd in toys and jewelry [3].

Cd accumulates in the body, mainly in the kidney, and is excreted very slowly, at a rate of about ~0.001% per day via the urine [4]. Exposure to Cd is typically measured in either blood or urine. Urine Cd, with a half-life of 30 years, reflects accumulated Cd body burden [4], while blood Cd, with a half-life of three to four months, reflects more recent exposure [5]. Cd has been associated with a numerous adverse health effects, including impaired kidney function, diabetes, hypertension, osteoporosis, and cancers in adults [2,4-7], as well as immunosuppression, neurotoxicity, and neurodevelopmental problems in children [8-11].

Iron deficiency (ID) is the most common and widespread nutritional disorder in the world [12]. It is believed to affect over 300 million pre-school and school-aged children [13]. While highly prevalent in developing countries, it is still a problem in many industrialized countries [14,15]. ID is associated with a multitude of health problems in children and adults [16]. In infants and young children, specifically, ID has been associated with poorer physical and mental development, and reduced cognitive function [1,13,17-20].

Dietary Cd absorption is thought to increase when iron stores are depleted. Studies in animals have demonstrated associations between ID and increased uptake of Cd via the diet [21-23]. Several studies in human adults have also found inverse associations between indicators of iron status and Cd levels. Three studies found that Cd in blood was inversely associated with serum ferritin (SF) levels in women [24-26], as well as both sexes [27]. Others have found increased urine Cd levels in pregnant women with poor iron status, as defined by SF [28] or the ratio of transferrin receptor to SF [29]. A recent study also found inverse associations between total body iron and both urine and blood Cd in women [30]. We were only able to identify one study that examined this association in children. Turgut and colleagues examined blood lead (Pb), copper (Cu), Cd, SF, and hemoglobin (Hgb) levels in a modest sample of children in Turkey (n = 256), where ID and heavy metal exposures are common. They found significantly higher levels of all three metals in children with iron deficiency anemia (IDA) but not with ID alone [31].

The aim of this study was to determine the extent to which Cd exposure and iron status are associated in a relatively low-exposed population of U.S. children.

## Methods

### Ethics statement

The National Health and Nutrition Examination Surveys (NHANES) is a publicly available data set administered by the National Center for Health Statistics (NCHS), part of the U.S. Centers for Disease Control and Prevention. Written informed consent (parents of children <18 years old or participants ≥18 years old) and assent (children ≥12 years old), consistent with approval by the NCHS Institutional Review Board, were obtained prior to participation in NHANES.

### Study population

Data were from the NHANES 1999/2000 and 2001/02 study cycles [32,33]. NHANES is a cross-sectional study designed to be representative of the health and diet of the non-institutionalized U.S. population. For the present study, 6856 children, 19 years of age or younger, were eligible for the blood Cd portion of the study. A subset of those (n = 1840) had urine Cd measurements available. Children with missing data for age, poverty income ratio, and serum cotinine were excluded, decreasing the eligible sample size to 6039 (n = 1639 for urine subsample). Additional exclusions were made to eliminate the influence of inflammation or infection on the iron measurements (see Iron status section of Methods for further explanation), decreasing the final sample size to 5224 subjects (n = 1430 for urine subsample).

### Cadmium biomarkers

Blood and urine Cd were measured at the Environmental Health Laboratory of the National Center for Environmental Health (Atlanta, GA, USA). NHANES laboratory protocols employ extensive quality assurance/quality control procedures, the details of which are described elsewhere [34-37].

Blood Cd was measured in whole blood using a multi-element atomic absorption spectrometer with Zeeman background correction [34,35]. National Institute of Standards and Technology whole blood standard reference materials were used for external calibration. The inter-assay coefficients of variation for blood Cd ranged from 4.1% to 7.3% [34,35]. The limit of detection (LOD) for blood Cd was 0.3 μg/L. Values below the LOD were assigned a value 0.2 μg/L (n = 3204), which was equal to the LOD divided by the square root of two [38,39]. Due to the large number of children with values below the LOD, we categorized blood Cd into three categories for analysis as the independent variable in the blood Cd models: <LOD, 0.3-0.4, ≥0.5 μg/L.

Urine Cd was measured in spot urine samples from a subset of NHANES participants aged six years and older, using inductively coupled plasma mass spectrometry [36,37]. National Institute of Standards and Technology urine standard reference materials were used for external calibration. The inter-assay coefficients of variation for urine Cd ranged from 1.3% to 6.7% [36,37]. The LOD for urine Cd was 0.06 ng/mL. Measurements below the LOD were assigned a value of 0.04 ng/mL (n = 71), which was equal to the LOD divided by square root of two [40,41]. Urine Cd was also manually corrected for molybdenum oxide interference [40,41]. Creatinine (Cr)-adjusted urine Cd (μg/g Cr) was created by dividing urine Cd (ng/mL) by urinary creatinine (UCr) (ng/mL). Cr-adjusted values are expressed here as ng/g Cr. The urine Cd distribution was right-skewed, necessitating a natural-log transformation, but there were urine Cd values = 0 (n = 40), which would have been lost if a natural-log transformation was used. Therefore, we categorized urine Cd into tertiles for analysis as the independent variable in the urine Cd models: ≤0.1, >0.1-0.2, >0.2 ng/mL and ≤0.7, >0.7-1.3, >1.3 ng/g Cr.

### Iron status

Details regarding the quantification of iron status indicators are described elsewhere [38,39,42,43]. Briefly, serum ferritin (SF) was measured using a single-incubation two-site immunoradiometric assay from Bio-Rad Laboratories (QuantImmune Ferritin IRMA kit) [38,39]. Free erythrocyte protoporphyrin (FEP) was quantitatively determined by molecular fluorometry using a spectrofluorometer [39,42]. The final FEP value was expressed as μg/dL of packed red blood cells (RBC). Percent transferrin saturation (TSAT) was calculated by dividing serum iron by total iron-binding capacity (TIBC) and multiplying by 100. Iron and TIBC were measured on an Alpkem Flow Solutions IV (rapid-flow analysis) system [39,43]. Hemoglobin (Hgb) was quantified using a Beckman Coulter MAXM instrument [44,45].

Iron deficiency (ID) was defined as ≥2 of 3 abnormal iron indicators (SF, FEP, TSAT), based on age-specific norms (Table 1) [46]. Children with a white blood cell count above 10×10^9^ cells/L or a C-Reactive Protein level greater than 6 mg/L were excluded from the analysis, due to concerns about the effects of infection and/or inflammation on some iron measurements [47-50]. Iron deficiency anemia (IDA) was defined as ID plus low Hgb, based on age and sex specific norms (Table 1) [46].

**Table 1:** Cut-off values for abnormal iron indicators and Hgb used to define ID and IDA

**Age (years)**	**SF (μg/L)**	**TSAT (%)**	**FEP (μg/dL)**	**Hgb (g/dL)**	
				**Girls**	**Boys**
3-5	<10	<12	>70	<11.2	<11.2
6-11	<12	<14	>70	<11.8	<11.8
12-15	<12	<14	>70	<11.9	<12.6
16-19	<12	<15	>70	<12.0	<13.6

### Covariates

Demographic information was obtained by self-report using computer-assisted personal interviews [51,52]. Gender was categorized as male or female. Continuous age, in years, was calculated using age, in months, at the time of the NHANES physical examination. Race/ethnicity was categorized into five groups: Mexican American, other Hispanic, Non-Hispanic Black (NHB), Other/Multi-Racial, and Non-Hispanic White (NHW). Poverty income ratio (PIR), created by the Department of Health and Human Services to quantify the ratio of family income to poverty [53], was categorized into quartiles <1.00, 1.00-1.80, 1.81-3.53, ≥3.54. These quartiles roughly represent categories for below poverty level, low-, middle-, and high-income [53]. Serum cotinine was measured by isotope dilution-high performance liquid chromatography/atmospheric pressure chemical ionization tandem mass spectrometry [38,39]. We categorized cotinine into <1, <1-10, and >10 ng/mL, to represent little or no exposure to secondhand smoke (SHS), high exposure to SHS, and likely smokers, respectively [54]. UCr, used to account for urine dilution, was measured using a modified Jaffé rate reaction and a Beckman Instruments CX3 analyzer [40,41]. UCr was used as a continuous covariate in models with urine Cd or as the denominator for calculating ng Cd/g Cr for urine samples.

### Statistical analysis

SAS version 9.3 (SAS Institute, Cary, NC) was used for all analyses. NHANES utilizes a complex, multistage design, in which certain subpopulations are oversampled. Sample weights can then be used to achieve results that are generalizable to the U.S. population. However, when factors employed in the formation of the sample weights, such as gender, age, race/ethnicity, or PIR, are included as covariates in the analysis, a weighted approach can be inefficient [55]. Here, we opted not to use sampling weights, instead analyzing the data as a cross-sectional cohort analysis.

Descriptive statistics, frequencies, and correlations for all variables of interest were examined. Based on the distribution of the variables of interest, logistic regression was used to evaluate associations between ID, IDA, or binary iron indicators (abnormal or normal) and categories of blood or urine Cd exposure. Crude and adjusted (for gender, age, race/ethnicity, PIR, and serum cotinine) models were examined for all relationships of interest. Urine Cd models were run two ways: with UCr as a covariate in the model [56] and using Cr-adjusted urine Cd concentration as the exposure.

Sensitivity analyses were completed to check the robustness of the analysis. Blood and urine Cd models were run after excluding children with serum cotinine >10 ng/mL (likely smokers). Blood Cd models were run for the subset of children with both urine and blood Cd data. Continuous iron indicators (natural-log[SF], natural-log[FEP], and TSAT) were used in logistic regression models of binary blood Cd (≥LOD vs. <LOD). Continuous urine Cd was used in logistic regression models of ID, IDA, low SF, high FEP, and low TSAT. Natural-log[SF], natural-log[FEP], and TSAT were used in linear regression models of continuous urine Cd/g Cr.

Age and sex-specific analyses of the blood Cd/iron status associations were also conducted. Several methods were used, including stratified frequencies, stratified models, and interaction terms. Analysis groups included boys or girls ages 3–11, 12–15, or 16–19 years, for a total of six groups. These groups were chosen using the same age/sex categories as the iron indicator cut-offs [46], though here we combined the two youngest age groups for simplicity. Crude and adjusted stratified models were run as before. However, instead of three categories of blood Cd, binary blood Cd (<LOD or ≥ LOD) was used to reduce the likelihood of having analysis groups with very low numbers. In an effort to be more parsimonious, other covariates were not included in these analyses.

## Results

There were no significant differences in demographics between those excluded from the analysis due to missing data and those included, with the exception of age. The excluded group was slightly younger, since serum cotinine was only available in children three years of age and older. The mean, 95th percentile, and maximum for blood Cd were 0.30, 0.40, and 4.00 μg/L, respectively. For urine Cd, the mean, 95th percentile, and maximum were 0.16, 0.32, and 1.80 ng/mL, respectively. The mean, 95th percentile, and maximum for Cr-adjusted urine Cd were 1.19, 2.14, and 14.49 ng/g Cr, respectively. Additional population characteristics can be found in Table 2. The prevalence of ID was 7.0% (n = 365); IDA was observed in 1.5% (n = 77). 29.2% of children (n = 1603) had at least one abnormal iron indicator: 8.1% (n = 422) had low SF, 10.8% (n = 563) had high FEP, 17.6% (n = 920) had low TSAT, and 5.2% (n = 269) had low Hgb. Table 3 shows frequencies for ID, IDA, and abnormal iron indicators. Blood and urine Cd were weakly but significantly correlated; Spearman rank ρ = 0.25, p < 0.0001 (ρ = 0.20, p < 0.0001 for Cr-adjusted urine Cd). Overall, ID was most common in girls, among Mexican American children, and was found to increase in adolescence. Blood Cd increased with serum cotinine (Spearman rank ρ = 0.20, p < 0.0001) and also with age (Spearman rank ρ = 0.32, p < 0.0001). Blood Cd was highest in boys, Mexican American, and NHB children. Urine Cd was also highest in NHB and Mexican American children, and also increased with age (Spearman rank ρ = 0.27, p < 0.0001).

**Table 2:** Characteristics of the study population

	**N (%)**
**Gender (F)**	2502 (47.9%)
**Age (years)**	
3-5	538 (10.3%)
6-11	1429 (27.4%)
12-15	1695 (32.5%)
16-19	1562 (29.9%)
**Race/ethnicity**	
Mexican American	1791 (34.3%)
Other Hispanic	243 (4.7%)
Non-Hispanic Black	1582 (30.3%)
Other/Multi Racial	213 (4.1%)
Non-Hispanic White	1395 (26.7%)
**Poverty Index Ratio (PIR)**	
<1.00 (below poverty level)	1739 (33.3%)
1.00-1.80 (low income)	1260 (24.1%)
1.81-3.53 (middle income)	1220 (23.4%)
3.54-5.00 (high income)	1005 (19.2%)
**Serum cotinine (ng/mL)**	
>10 (likely smoker)	428 (8.2%)
1-10 (high SHS exposure)	714 (13.7%)
<1 (no/low SHS exposure)	4082 (78.1%)

**Table 3:** Frequencies of ID, IDA, and abnormal iron indicators

**Exposure**		**ID**	**IDA**	**Low SF**	**High FEP**	**Low TSAT**
	**N**	**N (%)**	**N (%)**	**N (%)**	**N (%)**	**N (%)**
**Blood Cd (ug/L)**						
High (≥0.5)	512	58 (11.3%)	17 (3.3%)	69 (13.5%)	62 (12.1%)	106 (20.7%)
Med. (0.3-0.4)	1508	151 (10.0%)	32 (2.1%)	184 (12.2%)	186 (12.3%)	291 (19.3%)
Low (<LOD)	3204	156 (4.9%)	28 (0.9%)	169 (5.3%)	315 (9.8%)	523 (16.3%)
Total	5224	365 (7.0%)	77 (1.5%)	422 (8.1%)	563 (10.8%)	920 (17.6%)
**Urinary Cd (ng/mL)**						
High (>0.2)	471	40 (8.5%)	10 (2.1%)	88 (18.7%)	57 (12.1%)	48 (10.2%)
Med. (>0.1 to 0.2)	487	33 (6.8%)	6 (1.2%)	93 (19.1%)	54 (11.1%)	40 (8.2%)
Low (≤0.1)	472	29 (6.1%)	5 (1.1%)	91 (19.3%)	51 (10.8%)	33 (7.0%)
Total	1430	102 (7.1%)	21 (1.5%)	272 (19.0%)	162 (11.3%)	121 (8.5%)
**Urinary Cd (ng/g Cr)**						
High (>1.3)	467	45 (9.6%)	7 (1.5%)	102 (21.8%)	62 (13.3%)	52 (11.1%)
Med. (>0.7 to 1.3)	489	30 (6.3%)	6 (1.2%)	80 (16.4%)	54 (11.0%)	35 (7.2%)
Low (≤0.7)	474	27 (5.7%)	8 (1.7%)	90 (19.0%)	46 (9.7%)	34 (7.2%)
Total	1430	102 (7.1%)	21 (1.5%)	272 (19.0%)	162 (11.3%)	121 (8.5%)

Crude and adjusted logistic regression results can be found in Table 4. Odds of ID, IDA, low SF, and low TSAT were associated with increasing category of blood Cd. Adjusted odds ratios (ORs) and 95% confidence intervals (95% CI) for high blood Cd (≥0.5 μg/L) versus < LOD were 1.74 (1.30-2.34), 4.02 (1.92-8.41), 4.08 (2.36-5.89), and 1.78 (1.32-2.39), for IDA, ID, low SF, and low TSAT, respectively. The p-values for trend were also statistically significant for all four models. Results for urine Cd were less striking, though crude odds of ID and low SF were associated with increasing category of urine Cd (per gram Cr). However, these associations for urine Cd did not remain statistically significant after adjustment for other covariates.

**Table 4:** **Logistic regression model results**^**a**^

		**Blood Cd**		**Urinary Cd/g Cr**		**Urinary Cd**	
**Outcome**	**Exposure**	**OR (95% CI)**		**OR (95% CI)**		**OR (95% CI)**	
		**Crude**	**Adjusted**^**b**^	**Crude**	**Adjusted**^**b**^	**Crude**^**c**^	**Adjusted**^**d**^
**ID**	High vs. low Cd	2.50 (1.82-3.43)	2.78 (1.87-4.12)	1.77 (1.08-2.90)	1.37 (0.81-2.34)	1.64 (0.91-2.97)	1.21 (0.64-2.28)
	Med. vs. low Cd	2.17 (1.72-2.74)	1.90 (1.48-2.43)	1.08 (0.63-1.85)	1.01 (0.58-1.77)	1.17 (0.69-2.00)	1.06 (0.61-1.86)
	p-trend	<0.0001	<0.0001	0.02	0.22	0.10	0.56
**IDA**	High vs. low Cd	3.90 (2.11-7.17)	4.02 (1.92-8.41)	1.00 (0.46-2.19)	0.79 (0.35-0.75)	1.78 (0.55-5.73)	0.83 (0.24-2.82)
	Med. vs. low Cd	2.46 (1.48-4.10)	2.00 (1.17-3.42)	0.82 (0.35-1.89)	0.80 (0.34-1.89)	1.10 (0.35-3.48)	0.83 (0.25-2.77)
	p-trend	<0.0001	0.0001	0.86	0.48	0.33	0.77
**Low SF**	High vs. low Cd	2.80 (2.08-3.77)	4.08 (2.36-5.89)	1.62 (1.03-2.55)	1.42 (0.88-2.29)	1.91 (1.10-3.31)	1.63 (0.91-2.92)
	Med. vs. low Cd	2.50 (2.02-3.11)	2.38 (1.89-3.00)	1.00 (0.61-1.63)	0.99 (0.59-1.63)	1.30 (0.80-2.13)	1.28 (0.76-2.14)
	p-trend	<0.0001	<0.0001	0.03	0.14	0.02	0.1
**High FEP**	High vs. low Cd	1.26 (0.95-1.69)	1.36 (0.95-1.95)	1.42 (0.95-2.14)	1.02 (0.66-1.58)	1.47 (0.91-2.38)	1.12 (0.67-1.88)
	Med. vs. low Cd	1.29 (1.06-1.56)	1.07 (0.88-1.32)	1.16 (0.76-1.75)	0.99 (0.64-1.53)	1.14 (0.75-1.73)	0.99 (0.64-1.54)
	p-trend	0.013	0.133	0.09	0.91	0.19	0.67
**Low TSAT**	High vs. low Cd	1.34 (1.06-1.69)	1.74 (1.30-2.34)	1.19 (0.87-1.64)	1.07 (0.77-1.49)	1.12 (0.76-1.65)	0.94 (0.62-1.41)
	Med. vs. low Cd	1.23 (1.05-1.44)	1.26 (1.07-1.48)	0.84 (0.60-1.16)	0.82 (0.58-1.15)	1.05 (0.75-1.46)	1.02 (0.72-1.43)
	p-trend	0.002	<0.0001	0.27	0.67	0.57	0.76

^a^Crude and adjusted odds of ID, IDA, low SF, high FEP, and low TSAT for children with high and medium Cd versus low Cd.

^b^Models adjusted for gender, age, race/ethnicity, PIR, and serum cotinine.

^c^Models adjusted for UCr only.

^d^Models adjusted for gender, age, race/ethnicity, PIR, serum cotinine, and UCr.

Sensitivity analyses confirmed the robustness of the blood Cd results. The exclusion of likely smokers did not significantly change the associations between blood Cd and increased odds of ID, IDA, low SF, and low TSAT (Additional file 1: Table S1). Analysis of the blood Cd associations in the subset of children with both urine and blood Cd data also did not meaningfully affect the results (Additional file 1: Table S2). Replacement of categorical variables with continuous ones also produced similar trends, statistical significance, and directions of association as the categorical analyses (Additional file 1: Table S3). The only exception was that the low TSAT/blood Cd relationship lost statistical significance when continuous TSAT was used (Additional file 1: Table S3).

Age and sex-stratified frequencies as well as stratified model results are shown in Table 5. Girls, 12–19 years old, had higher frequencies of ID, low SF, high FEP, and low TSAT than boys of the same age. Compared to boys, girls aged 3–11 years had significantly higher odds of having low SF if they had blood Cd ≥ LOD; OR (95% CI) = 2.03 (1.08-3.83). Girls aged 12–15 years with blood Cd ≥ LOD had significantly higher odds of having ID, low SF, and low TSAT; ORs (95% CI) = 2.13 (1.39-3.26), 2.63 (1.77-3.89), and 1.46 (1.05-2.03), respectively. Girls aged 16–19 years had increased odds of ID and abnormal iron indicators for all iron indicators; ORs (95% CI) = 2.36 (1.58-3.63), 3.12 (2.08-4.68), 1.62 (1.11-2.35), and 1.55 (1.10-2.16), for ID, low SF, high FEP, and low TSAT, respectively. Boys aged 12–15 years with high blood Cd also had increased odds of having ID and low SF; ORs (95% CI) = 2.35 (1.21-4.58) and 2.62 (1.30-4.93), respectively. The ID model for 16- to 19-year-old boys did not converge due to very low numbers of ID.

**Table 5:** **Age and sex stratified frequencies and logistic regression model results**^**a**^

		**ID**		**Low SF**		**High FEP**		**Low TSAT**	
**Stratification group**	**N**	**N (%)**	**OR (95% CI)**	**N (%)**	**OR (95% CI)**	**N (%)**	**OR (95% CI)**	**N (%)**	**OR (95% CI)**
Girls 3–11 yrs	945	41 (4.3%)	1.62 (0.83-3.20)	44 (4.7%)	2.03 (1.08-3.83)	92 (9.7%)	1.31 (0.81-2.13)	167 (17.7%)	0.93 (0.62-1.39)
Boys 3–11 yrs	1022	36 (3.5%)	0.97 (0.45-2.09)	45 (4.4%)	1.19 (0.62-2.31)	78 (7.6%)	0.93 (0.55-1.60)	182 (17.8%)	1.25 (0.87-1.78)
Girls 12–15 yrs	859	97 (11.3%)	2.13 (1.39-3.26)	119 (13.9%)	2.63 (1.77-3.89)	136 (15.8%)	1.29 (0.89-1.88)	194 (22.6%)	1.46 (1.05-2.03)
Boys 12–15 yrs	836	38 (4.6%)	2.35 (1.21-4.58)	43 (5.1%)	2.62 (1.39-4.93)	65 (7.8%)	0.92 (0.54-1.54)	102 (12.2%)	1.19 (0.78-1.81)
Girls 16–19 yrs	698	145 (20.8%)	2.36 (1.58-3.63)	161 (23.1%)	3.12 (2.08-4.68)	155 (22.2%)	1.62 (1.11-2.35)	208 (29.8%)	1.55 (1.10-2.16)
Boys 16–19 yrs	864	8 (0.9%)	^b^	10 (1.2%)	1.7 (0.44-6.62)	37 (4.3%)	0.95 (0.49-1.84)	67 (7.8%)	1.65 (0.96-2.81)

^a^Unadjusted odds of ID, low SF, high FEP, and low TSAT for children with blood Cd ≥ LOD versus blood Cd < LOD.

^b^Model did not converge.

## Discussion

With increasing levels of blood Cd, the odds of ID, IDA, low SF, and low TSAT also increased. These associations remained significant, regardless of whether active smokers were excluded, the dataset was restricted to a smaller number of participants, categorical or continuous iron indicators were used, and exposure and outcome were reversed. These relations were not observed for urine Cd in models adjusted for relevant covariates. Girls, 12–19 years old, had higher frequencies of ID, low SF, high FEP and low TSAT than boys of the same age. Young girls had increased odds of low SF with high blood Cd. Boys and girls, ages 12–15, had increased odds of ID and low SF with high blood Cd, and 16- to 19-year-old girls with high blood Cd had increased odds of ID, low SF, high FEP, and low TSAT.

The results for blood Cd in this study are consistent with previous epidemiological findings, all of which found a significant inverse associations between varying indicators of iron status and blood Cd levels [24-27,30,31]. The urine Cd results, however, were not significant after adjustment for covariates. Three previous studies have found significant associations between iron levels and urine Cd [28-30], though all were in adults, and urine Cd levels were considerably higher (urine Cd means of 242 ng/g Cr [30] and 0.31 μg/L [29], compared to our reported means of 1.19 ng/g Cr and 0.16 μg/L). We found that urine Cd was correlated with age (Spearman rank ρ = 0.27, p < 0.0001), consistent with Cd accumulating in the kidney over a time. Since Cd accumulates in the kidney, it is possible that our study population of children was too young to have urine Cd concentrations high enough detect a clear association. Alternatively, urine Cd may not be an ideal biomarker when considering the association of Cd with current iron status, since urine Cd represents a more long-term measure of Cd exposure [4]. While indicators used to determine iron status are fairly stable on a day-to-day basis [57,58], they change markedly with development over longer periods of time and with the onset of puberty. Blood Cd, a measure of more recent Cd exposure [5], may therefore be a more appropriate biomarker for assessing relations with iron status.

To our knowledge, sex and age-specific results have not been reported prior to this analysis, as the majority of related studies to date have been only among adult females. Still, our finding that older girls are the most likely group to have ID or have abnormal iron indicators is not surprising, given that girls who have reached menarche are more likely to be ID than younger or less mature girls [15,59]. The significant associations between blood Cd and ID and low SF observed for boys aged 12–15, were somewhat surprising. This is not a group commonly studied in the ID field and we could find no precedent to which we could compare these findings. The results require replication, since the number of 12- to 15-year-old boys with ID and Cd ≥ LOD was small (n = 23).

Studies of Cd exposure and iron status among children are limited. The only study we could identify compared blood Cd levels in healthy, ID, and IDA children in Turkey [31]. ID was defined as low SF levels and IDA as low Hgb, in addition to low SF. The investigators found significantly higher levels of blood Cd in children with IDA, as compared to healthy children, but did not find a statistically significant difference in Cd levels when comparing ID children with healthy children, using ANOVA [31]. Our results extend the Turkey study by using a multi-indicator model for defining ID, more sophisticated data analyses, and a much larger sample size (n = 5224 compared to 256).

Although there has been little research on ID and Cd in children, several studies have examined similar relationships between ID and blood Pb and have found significant inverse associations between Pb and ID [60-62]. It is possible that Pb and Cd, both divalent metals, may share a similar mechanism of uptake. One such proposed mechanism for the apparent increase of divalent metals in the presence of ID is increased expression of divalent metal transporter 1 (DMT1) when iron status is compromised [21,63]. Higher levels of DMT1, in theory, help the body to absorb more iron from the intestine. If Cd or other divalent metals are present in the diet, upregulation of DMT1 due to ID may also contribute to the increased uptake of toxic heavy metals.

The present analysis has several noteworthy strengths. To our knowledge, this is the first study to demonstrate an association between ID and blood Cd in children in the U.S. We used the multiple indicator ferritin model to determine iron status, which takes into account the levels of three iron indicators: SF, FEP, and TSAT. This represents an improvement over some previous studies, many which have used SF levels alone to determine iron status [24,25,27,31]. Recently, some researchers have advocated the use of another multi-indicator model for iron status, “body iron”, which yields a single parameter ranging from iron excess to iron deficit [47]. We were unable to use the body iron model for this study, since serum transferrin receptor, which is needed to calculate body iron, is unavailable in NHANES prior to 2003. We chose NHANES 1999/2000 and 2001/02 cycles because we wanted to assess the relationship between Cd and ID in children of both sexes and all age ranges. In 2003, NHANES stopped taking iron measurements for children of all ages and limited their analyses to children 1–5 years and females 12 years and older. Therefore, for our analysis we were limited to just the two early cycles of continuous NHANES. The primary limitation to using SF alone, or as part of the ferritin model, is the possible influence of infection or chronic inflammation on SF levels. To address this issue, we excluded children with signs of infection or inflammation, as indicated by a high white blood cell count or high C-reactive protein levels. In an effort to further improve the validity of our findings, we also conducted several sensitivity analyses, which revealed our findings were robust. Finally, we had a large sample size (n = 5224) and looked at the entire age range of children (with the exception of 1 and 2 year olds who were excluded due to missing serum cotinine values), which had not been examined before.

As with all cross-sectional analyses, this study is limited in that we cannot demonstrate causation or temporality. We do not know if poor iron status led to increased uptake of Cd or higher concentrations of Cd decreased iron absorption or storage. Additionally, we do not have any information regarding individual genetic susceptibility, since genetic polymorphisms of iron-related genes can be an important factor in the uptake of heavy metals, such as Cd and Pb [28,64-67]. Finally, future studies should include infants, who were excluded here due to missing data.

## Conclusions

In conclusion, in U.S. children aged 3–19 years, odds of ID and IDA increased with blood Cd, a biomarker of recent Cd exposure. Odds of low SF and low TSAT were also found to increase with blood Cd. Sex and age-specific analyses revealed that odds of ID, IDA, and abnormal iron indicators were highest in adolescent females.

Given their shared neurotoxic effects in children, and that many children live in areas with high burdens of both ID and heavy metals, more research into the complex relationships between nutrient deficiencies and environmental exposures is vital.

## Abbreviations

Cd: Cadmium; ID: Iron deficiency; IDA: Iron deficiency anemia; SF: Serum ferritin; FEP: Free erythrocyte protoporphyrin; TSAT: Percent transferrin saturation; RBC: Packed red blood cells; UCr: Urinary creatinine; NHANES: National health and nutrition examination survey; OR: Odds ratio; CI: Confidence interval; LOD: Limit of detection.

## Competing interests

The authors declare that they have no competing interests.

## Authors’ contributions

MS carried out the analysis, interpreted the results, and drafted the manuscript. BL interpreted the results and contributed to drafting the manuscript. JM conceived the study, interpreted the results, and contributed to drafting the manuscript. All authors read and approved the final manuscript.

## Supplementary Material

Additional file 1: Table S1 Logistic regression model results with likely smokers excluded. **Table S2**: Blood Cd logistic regression results for subsample analysis. **Table S3**: Model results when continuous variables were used. Click here for file
