# Supplementary material for: Blood cadmium is elevated in iron deficient U.S. children: a cross-sectional study
Source: Environ Health. 2013 Dec 30;12:117. doi: 10.1186/1476-069X-12-117 (PMC3883480; doi:10.1186/1476-069X-12-117)
Supplement: Additional file 1: Table S1 — Logistic regression model results with likely smokers excluded. Table S2: Blood Cd logistic regression results for subsample analysis. Table S3: Model results when continuous variables were used. [file 1476-069X-12-117-S1.docx]

**Table S1**

Logistic regression model results with likely smokers excluded ^a^

|  |  | **Blood Cd** | | **Urinary Cd/ g Cr** | | **Urinary Cd** | |
| --- | --- | --- | --- | --- | --- | --- | --- |
| **Outcome** | **Exposure** | **OR (95% CI)** | | **OR (95% CI)** | | **OR (95% CI)** | |
|  |  | **Crude** | **Adjusted ^b^** | **Crude** | **Adjusted ^b^** | **Crude ^c^** | **Adjusted** ^d^ |
| **ID** | High vs. low Cd | 4.04 (2.75-5.94) | 2.90 (1.92-4.38) | 1.58 (0.95-2.64) | 1.23 (0.71-2.13) | 1.67 (0.90-3.12) | 1.22 (0.63-2.38) |
|  | Med. vs. low Cd | 2.26 (1.79-2.86) | 1.90 (1.48-2.44) | 0.92 (0.52-1.62) | 0.86 (0.48-1.54) | 1.15 (0.67-2.02) | 0.99 (0.55-1.79) |
|  | p-trend | <0.0001 | <0.0001 | 0.07 | 0.40 | 0.11 | 0.54 |
| **IDA** | High vs. low Cd | 5.84 (2.87-11.90) | 3.87 (1.79-8.37) | 1.11 (0.50-2.44) | 0.90 (0.40-2.01) | 2.30 (0.67-7.94) | 1.16 (0.31-4.32) |
|  | Med. vs. low Cd | 2.52 (1.51-4.22) | 1.97 (1.15-3.38) | 0.76 (0.30-1.91) | 0.75 (0.29-1.90) | 1.11 (0.32-3.95) | 0.81 (0.22-3.03) |
|  | p-trend | <0.0001 | 0.0003 | 0.99 | 0.65 | 0.18 | 0.78 |
| **Low SF** | High vs. low Cd | 4.92 (3.45-7.02) | 4.14 (2.83-6.06) | 1.39 (0.87-2.21) | 1.21 (0.74-1.98) | 1.66 (0.94-2.95) | 1.41 (0.77-2.58) |
|  | Med. vs. low Cd | 2.60 (2.08-3.24) | 2.36 (1.87-2.98) | 0.84 (0.50-1.40) | 0.82 (0.49-1.39) | 1.18 (0.71-1.97) | 1.12 (0.66-1.91) |
|  | p-trend | <0.0001 | <0.0001 | 0.15 | 0.40 | 0.08 | 0.26 |
| **High FEP** | High vs. low Cd | 1.94 (1.34-2.79) | 1.31 (0.89-1.93) | 1.42 (0.94-2.16) | 1.03 (0.66-1.60) | 1.58 (0.96-2.59) | 1.21 (0.71-2.05) |
|  | Med. vs. low Cd | 1.33 (1.10-1.62) | 1.07 (0.87-1.31) | 1.08 (0.70-1.67) | 0.92 (0.58-1.45) | 1.13 (0.73-1.74) | 0.95 (0.60-1.51) |
|  | p-trend | <0.0001 | 0.211 | 0.09 | 0.88 | 0.08 | 0.51 |
| **Low TSAT** | High vs. low Cd | 1.99 (1.46-2.70) | 2.01 (1.46-2.77) | 1.13 (0.80-1.58) | 1.04 (0.73-1.47) | 1.11 (0.74-1.67) | 0.95 (0.62-1.47) |
|  | Med. vs. low Cd | 1.24 (1.05-1.46) | 1.25 (1.05-1.48) | 0.81 (0.57-1.15) | 0.82 (0.57-1.17) | 1.01 (0.71-1.43) | 0.98 (0.68-1.41) |
|  | p-trend | <0.0001 | <0.0001 | 0.46 | 0.81 | 0.64 | 0.83 |

^a^ - Crude and adjusted odds of ID, IDA, low SF, high FEP, and low TSAT for children with high and medium Cd versus low Cd, with likely smokers (serum cotinine >10 ng/mL) excluded from the analysis. N=4796 for the blood Cd analyses and N=1298 for the urine Cd analyses.

^b^ - Models adjusted for gender, age, race/ethnicity, PIR, and serum cotinine (SHS versus no SHS)

^c^ - Models adjusted for UCr only

^d^ - Models adjusted for gender, age, race/ethnicity, PIR, serum cotinine, and UCr

**Table S2**

Blood Cd logistic regression results for subsample analysis ^a^

| **Outcome** | **Exposure** | **OR (95% CI)** | |
| --- | --- | --- | --- |
|  | **Blood Cd** | **Crude** | **Adjusted** ^b^ |
| **ID** | High vs. low Cd | 3.85 (2.22-6.67) | 4.53 (2.22-9.23) |
|  | Med. vs. low Cd | 2.55 (1.61-4.03) | 2.30 (1.41-3.74) |
|  | p-trend | <0.0001 | <0.0001 |
| **IDA** | High vs. low Cd | 3.85 (1.07-13.80) | 4.93 (1.12-21.73) |
|  | Med. vs. low Cd | 4.14 (1.52-11.28) | 3.04 (1.06-8.75) |
|  | p-trend | 0.01 | 0.02 |
| **Low SF** | High vs. low Cd | 3.73 (2.24-6.20) | 5.94 (3.09-11.42) |
|  | Med. vs. low Cd | 2.37 (1.56-3.63) | 2.38 (1.52-3.73) |
|  | p-trend | <0.0001 | <0.0001 |
| **High FEP** | High vs. low Cd | 1.22 (0.72-2.07) | 1.13 (0.57-2.23) |
|  | Med. vs. low Cd | 1.35 (0.94-1.94) | 1.08 (0.73-1.59) |
|  | p-trend | 0.18 | 0.65 |
| **Low TSAT** | High vs. low Cd | 1.57 (1.05-2.34) | 2.00 (1.17-3.42) |
|  | Med. vs. low Cd | 1.14 (0.84-1.54) | 1.22 (0.89-1.68) |
|  | p-trend | 0.03 | 0.02 |

^a^ - Crude and adjusted odds of ID, IDA, low SF, high FEP, and low TSAT for children with high and medium blood Cd versus low blood Cd. N=1430 (subset of children with both urine and blood Cd measurements)

^b^ - Models adjusted for gender, age, race/ethnicity, PIR, and serum cotinine

**Table S3**

Models results when continuous variables were used

| **Outcome** | **Exposure** | **OR (95% CI)** | | | | | |
| --- | --- | --- | --- | --- | --- | --- | --- |
|  |  | **Crude** | | | **Adjusted ^a^** | | |
| **Blood Cd ≥LOD vs. <LOD** | Ln(SF) | 0.87 (0.80-0.94) | | | 0.66 (0.60-0.72) | | |
|  | Ln(FEP) | 1.30 (1.10-1.55) | | | 1.14 (0.94-1.40) | | |
|  | TSAT | 1.01 (1.00-1.01) | | | 1 (0.99-1.00) | | |
| **ID** | Urine Cd/ g Cr | 3.83 (0.91-16.12) | | | 1.25 (0.24-6.64) | | |
|  | Urine Cd | 3.66 (1.10-12.20) | | | 1.77 (0.43-7.35) | | |
| **IDA** | Urine Cd/ g Cr | 2.08 (0.07-58.76) | | | 0.36 (0.003-50.58) | | |
|  | Urine Cd | 2.69 (0.84-5.71) | | | 1.53 (0.55-4.26) | | |
| **Low SF** | Urine Cd/ g Cr | 4.12 (1.07-15.79) | | | 2.00 (0.46-8.67) | | |
|  | Urine Cd | 4.62 (1.50-14.30) | | | 2.97 (0.88-10.00) | | |
| **High FEP** | Urine Cd/ g Cr | 3.00 (0.84-10.78) | | | 0.91 (0.21-3.87) | | |
|  | Urine Cd | 2.94 (0.98-8.79) | | | 1.42 (0.40-4.98) | | |
| **Low TSAT** | Urine Cd/ g Cr | 2.36 (0.77-7.26) | | | 1.48 (0.45-4.84) | | |
|  | Urine Cd | 2.20 (0.84-5.71) | | | 1.53 (0.55-4.26) | | |
| **Outcome** | **Exposure** | **Crude** | | | **Adjusted ^b^** | | |
|  |  | **ß** | **SE** | **p** | **ß** | **SE** | **p** |
| **Urine Cd/ g Cr** | Ln(SF) | -0.010 | 0.003 | 0.01 | -0.004 | 0.004 | 0.28 |
|  | Ln(FEP) | 0.026 | 0.008 | 0.002 | 0.007 | 0.009 | 0.43 |
|  | TSAT | -0.0001 | 0.0003 | 0.67 | 0.0002 | 0.0003 | 0.51 |

^a^ - Models adjusted for gender, age, race/ethnicity, PIR, and serum cotinine. Urine Cd (not urine Cd/ g Cr) models were additionally adjusted for UCr.

^b^ - Models adjusted for gender, age, race/ethnicity, PIR, and serum cotinine.
